# A smart learning ecosystem design for delivering Data-driven Thinking in STEM education

**DOI:** 10.1186/s40561-021-00153-y

**Published:** 2021-07-13

**Authors:** Francisco Benita, Darshan Virupaksha, Erik Wilhelm, Bige Tunçer

**Affiliations:** Singapore University of Technology and Design, 8 Somapah Road, Singapore, 487372 Singapore

**Keywords:** Data-driven thinking, STEM education, Internet of things, Experiential learning

## Abstract

This paper proposes an Internet of Things device (IoT)-based ecosystem that can be leveraged to provide children and adolescent students with STEM educational activities. Our framework is general and scalable, covering multi-stakeholder partnerships, learning outcomes, educational program design and technical architecture. We highlight the importance of bringing Data-driven Thinking to the core of the learning environment as it leads to collaborative learning experience and the development of specific STEM skills such as problem-finding and solving, cognitive, analytical thinking, spatial skills, mental manipulation of objects, organization, leadership, management, and so on. A successful case study in Singapore involving tens of thousands of students is presented.

## Introduction

In the light of the increasing digitalization of society, the rapid growth of Big Data, Internet of Things (IoT) or Artificial Intelligence applications has boosted the demand for experienced professionals in STEM (Science, Technology, Engineering, and Mathematics) areas. The hype associated with these applications has bring tremendous challenges and opportunities to STEM education. Various stakeholders within the educational context have proposed digital technologies such as IoT devices in the in- and out-of-school learning settings for children and adolescent students’ education (Ito et al., 2015). An important question is then how STEM education initiatives can adapt current trends of in- and out-of-school digital practices (Ning & Hu, 2012). Among the main challenges that need to be tackled are the adoption of new relationships between learners and teachers (Coccoli, Guercio, Maresca, & Stanganelli, 2014); the design of frameworks enabling assimilation of data-driven processes (Bielaczyc, 2006), and; the definition of digital strategies and education policies established to guide relevant stakeholders’ engagement (Lee, Zo, & Lee, 2014).

Many proposals on how STEM education shall evolve while adapting and adopting these new technologies can be found in the published literature. Some studies focused on bringing specific Computer Science contents into schools’ curricula (Buffum et al., 2014; Wing, 2006). Some others preferred more hands-on approaches using hardware components, such as single-board computers or microcontrollers, to offer practical experiences in schools (He, Ji, & Bobbie, 2017). On a higher level, some researchers have explored how new digital technologies can be leveraged in favor of active, informal, and collaborative learning (Freeman et al., 2014; Kitsantas & Dabbagh, 2012). The study of Fößl, Ebner, Schön, and Holzinger (2016), for instance, has shown that open education approaches using video support and mobile technology allow students to experience self-regulated learning and develop self-regulated learning strategies. Some other scholars have investigated how IoT can be exploited to augment learning experiences (Pei, Wang, Wang, & Li, 2013). All in all, the above-mentioned frameworks are ecosystems based on Smart Education (Lee, Zo, & Lee, 2014), wearable IoT devices in STEM education (Minerva, Biru, & Rotondi, 2015), and Computational Thinking (Wing, 2006).

Notable STEM education initiatives and learning ecosystems that took place over the past decade (Zhu, Yu, & Riezebos, 2016) are the Malaysian Smart School Implementation Plan (Malaysia), Intelligent Nation Master Plan (Singapore), Smart, multi-disciplinary student-centric education system (Australia), SMART (South Korea), New York’s Smart School (United States), SysTec (Finland) or Mohammed Bin Rashid Smart Learning Program (United Arab Emirates). However, most of them either summarize helpful guidelines and considerations for the design of smart learning environments or have been carried out on a pilot scale within few educational institutions.

Alternatively, this study aims at constructing a generalizable large-scale smart learning ecosystem that involves effective and efficient support (e.g., guidance, feedback, or tools) in the context of children and adolescent STEM education. Our framework is designed to foster critical thinking and problem solving by means of “Data-driven Thinking”. In a nutshell, our smart learning ecosystem i) promotes STEM education and Data-driven Thinking in a student-friendly manner with emphasis on collaborative and experiential learning; ii) integrates various stakeholders (such as pedagogical institutes, educators, funding bodies or research agencies) for a large-scale deployment, and; 3) is based on a wide range of (flexible) services and components, ranging from cloud computing to IoT devices, design of experiments and to analytic platforms. Moreover, we present a case study of about 100,000 students from 196 educational institutions (primary, secondary and pre-university) who participated in the Singapore’s National Science Experiment (NSE) over the period 2015–2017. The NSE initiative adopted our smart learning ecosystem with the aim of delivering Data-driven Thinking and educating children and adolescent students to be globally aware of STEM subjects. NSE is not only the largest IoT initiative worldwide to expose young students to environmental and mobility data but also to spur interest in STEM subjects.

## Background

### Smart education and wearable IoT devices

The concept of Smart Education is based on smart learning through, but not limited to, IoT devices and other Information and Communication Technologies (ICT), and it is closely related to the literature on Smart Cities (Lee, Zo, & Lee, 2014). More precisely, there are three main dimensions in Smart Education, namely, educational outcomes, ICT and organization.

Educational outcome is the most important dimension as it is the purpose of students upon which the smart education program is built. Whether the desired outcomes relate to the development of cognitive skills (cognitive self-organization, system thinking, logical and analytical thinking, etc.), digital literacy or smart life skills, pedagogical approaches should be carefully adopted. ICT and the technological architecture around it create flexible tools and well-adapted educational opportunities for learning. With the goal of enabling integrity, interactivity, social interaction tools and mobility, ICT blends elements of hardware, software and networks together with digital sensors and smart devices (Lara & Labrador, 2013). The organizational dimension comprises educational programs, forms of learning and principles of teaching (Tikhomirov, Dneprovskaya, & Yankovskaya, 2015).

### Computational Thinking and Data-driven Thinking

The seminal paper of Wing (2006) introduced the concept of Computational Thinking as a universally applicable attitude and skill set everyone should ideally learn and use. In her work, Jeannette Wing stressed the importance of such mindset to be developed in children for an effective learning in STEM education. Computational Thinking can be summarized as the thought process of formulating problems and their solutions so that they are represented in a form that can be effectively carried out by an information-processing agent. However, Grover and Pea (2013) highlight the definitional confusion concerning the term. This is, there is a number of perspectives and evolving definitions of Computational Thinking, together with a mix of different environments and tools believed to promote the above-mentioned mindset in the educational space. Data-driven Thinking is closely related to Computational Thinking as operations on data are expected to be computationally meaningful. Nevertheless, Data-driven Thinking refers to the thought process of addressing a problem (e.g., situation) and proposing solutions (e.g., actions) than can be efficiently formulated and backed by data (Tunçer, Benita, & Scandola, 2019). We also believe Data-driven Thinking to be an emerging trend within STEM education imposed by the ever-increasing ubiquitous use of data-driven processes in our society.

### The instructional design for Data-driven Thinking in STEM education

Project-based learning and collaborative learning have been shown to be effective strategies to engage young students in STEM education (Kelley & Knowles, 2016). Although there are many student-centred teaching and learning approaches, project-oriented problem-based learning is more useful in the context of delivering Data-driven Thinking in STEM education (Boss & Krauss, 2014). Project-oriented problem-based learning is one type of experiential learning (Kolb, 2014) with emphasis to transition students from passive observers to active participants. These experiential activities: (i) motivate and increase commitment among students; (ii) are problem-oriented and not subject-oriented; (iii) are based on learning process and methodologies designed to find solutions rather than recall knowledge, and; (iv) promote team work, social and communication skills. Particularly, collaborative learning (e.g., working in groups or teams) plays a key role in the instructional design as not only supports in- and out-of-school learning but also offers students a set of skills (negotiation, organization, leadership, management, etc.) needed for twenty-first century workers in STEM areas (Morrison, Roth McDuffie, & French, 2015).

Lastly, when the learning approach utilizes IoT devices and other assistive technologies, educational gaming environments are believed to have a unique ability to display information and knowledge. They are immersive and fun environments allowing freely interactions with little or no consequence. Recent research has revealed the potentially positive impact of gaming experience itself on STEM education among youth (Shank & Cotten, 2014; Sherry, 2015). Some (Meluso, Zheng, Spires, & Lester, 2012) argue that game-based learning provides intrinsically motivating environments enhancing STEM education. Some others (Aguilar, Holman, & Fishman, 2018) have shown they are cost-effective solutions at imparting desirable attributes (communication skills, adaptability or resourcefulness) which could be important for success in STEM related job environments.

## A smart learning ecosystem for enabling Data-driven Thinking in STEM education

### Stakeholders

By engaging stakeholders in the various stages of the educational initiative, the proposed framework is tasked to establish, organize, operate and maintain a smart learning ecosystem that promotes Data-driven Thinking in STEM. Our framework permits children and adolescent students to explore and experiment with data. It offers unique experiences enabling new perspectives, and, it provides opportunities to collaborate with others for their learning.

**Fig. 1:**
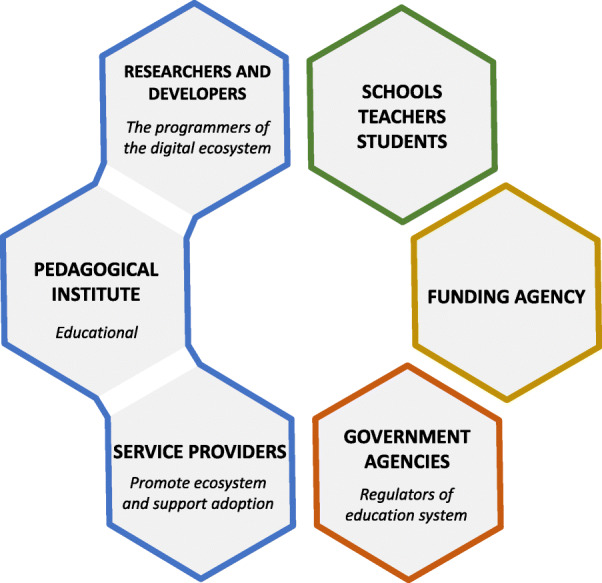
Stakeholders in the smart learning ecosystem that delivers Data-driven Thinking in STEM education

Figure 1 displays the stakeholders playing relevant roles in the development of the smart learning ecosystem. Schools, students, and teachers represent end users; thus, they are grouped together into the schema classification. Government agencies design and implement guidelines for the management, interaction and communication of educational institutes. Funding agencies look closely at the goals of educational projects and set stringent constraints on budget availability. Funding agencies and government institutions are represented in stand-alone hexagons as they are not always related institutions. It is expected that funding resources (or part of it) might come from private or non-governmental organizations. Finally, researchers and developers, pedagogical institutes, and service providers represent main operators of the smart learning ecosystem. These three partners are linked together as they build, execute and maintain ecosystem’s components.

#### Government agencies

Dialogue and exchange between educational leaders and policy personnel is the starting point in drawing smart learning programs. Local government authorities exert firm controls and can support STEM initiatives. Furthermore, in countries like China, India, United States or Russia, policy actions promoting influx and growth of STEM workforce in strategic areas have been taken for decades (Hira, 2010).

#### Funding agencies

After educational outcomes are clearly set out, funding provided by different entities, including government agencies, professional organizations, industries, and education institutions would help ensure meeting STEM program’s goals and objectives. The process is competitive, and it is important that the smart learning project aligns with the funding agency’s development agenda (Li et al., 2020).

#### Pedagogical institutes

Teaching and learning specialists shall have a major role in curating the structure and content of ecosystem. The specific responsibilities of pedagogical institutes include the following: designing, supervising and conducting learning activities, and; developing Data-driven Thinking-related curriculum pedagogical content knowledge and materials (e.g., blogs, websites, teaching materials, etc.). Additional tasks for these entities could be communicating and collaborating with software developers and content creation teams to ensure learning objectives remain consistent. Pedagogical institutes should also design, explore, propose and support the assessment of learning outcomes.

#### Schools, teachers and students

Schools serve as the physical and institutional backbone of the smart learning initiative. Schools’ facilities represent the reference location for teacher-student interaction. Thus, a smart learning ecosystem can take advantage of existing school’s physical IT resources and physical infrastructures such as laboratories, classrooms, and ICT infrastructure (the availability and quality of hardware, networks and connectivity within the school). With respect to teachers, they may require additional training on STEM-related challenges to deal with the adoption of the smart learning initiative. Teachers should work together with pedagogical institutes in actively engaged participatory activities tied to context-dependent learning needs.

#### Researchers and developers

They support students in their Data-driven Thinking endeavors by developing digital functionalities of the smart learning environment. The architecture and technology components that researcher and developer teams have to deal with are: (i) sensors and other sources of quality data; (ii) IoT cloud infrastructure, and; (iii) data processing and visualization functions (e.g., gamification). The next section elaborates the interactions of these three components.

#### Service providers

They are all those entities which are essential for maintaining operations of in- and out-of-school learning activities. In a simple manner, we can distinguish between basic services (such as those involving logistic), resource management, public relations, and communications.

## Data-driven Thinking in STEM education

**Fig. 2:**
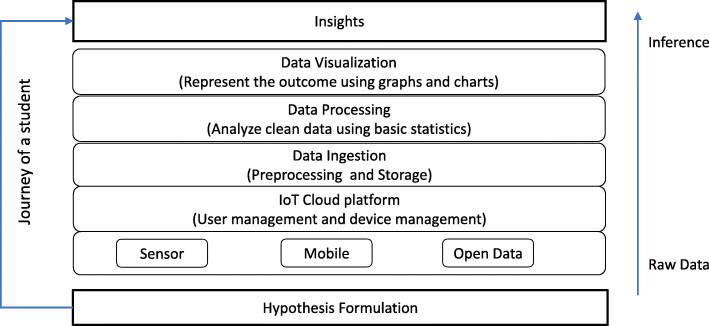
Data-driven Thinking and user journey

Our ecosystem is specially designed for learning through STEM-based Data-driven Thinking. It is built upon project-oriented problem-based learning and collaborative learning. Student’s journey through Data-driven Thinking is illustrated in Fig. 2 and the main stages of the learning process can be summarized as follows:

(i) Definition of research question and hypothesis formulation. To develop cognitive skills (cognitive logical and analytical thinking, see Wing (2006) and Grover and Pea (2013)) and get comprehensive insight into the usefulness of data to draw effective problem solutions.
(ii) Data collection from internal (smart learning ecosystem) and/or external sources (public databases, repositories, social media, etc.).
(iii) Data analysis and processing. Manual data manipulation (by students) and automated processing happening at cloud-level (by researchers and developers, see Fig. 1).
(iv) Data visualization. To transform text-based data into visually stimulating 2D or 3D charts, maps, graphs, or networks (Benita et al., 2020). Patterns, trends, and correlations can be distinguished and characterized with effective visualization techniques. Moreover, gaming environments can provide students with a diverse set of cognitive skills such as spatial skills or generating and manipulating mental representation of objects (Shank & Cotten, 2014; Sherry, 2015).
(v) Summary report. Where children and adolescent students can elaborate on important discovered insights and results. Here, students must explain and show how data served to test and validate their hypotheses.

## The National Science Experiment as case study

### General overview

The NSE was brought to life to instil a passion for STEM in young Singaporeans. This smart learning initiative involved more than 90,000 students from primary school (ages 7 to 12), secondary school (13 to 16), and pre-university (17 and 18) from 129 different schools around the country. To expose children and adolescent students to real-world science while encouraging them to think and work with the mindset of a STEM, it was adopted a Data-driven Thinking approach. Learning activities of the NSE journey, labelled as “Experiments”, were designed to guide users (e.g., *schools, teachers and students* from Fig. 1) across pre-selected tasks (designed by *pedagogical institutes*) while adopting a data-driven perspective. NSE offered two main types of experiential learning, namely: Data Collection and Big Data Challenge.

#### Data collection

It promoted literacy practices for conceptual and cognitive learning, and comprehension monitoring. This type of Experiment had strong emphasis on learning activities that involved the use of interactive data and its intuitive understanding. Data Collection did not require advanced STEM coursework on the one hand, and did not develop non-cognitive skills such as collaboration or problem solving on the other. Support and extra duties required from teachers were minimal and the duration of learning experiences was 1 week.

#### Big data challenge

Here, children and adolescent students experienced the whole cycle of Data-driven Thinking depicted in Fig. 2. It was designed into a collaborative and project-oriented problem based-learning. The exposure of students to Data-driven Thinking was higher but the total number of participants was lower than that envisioned in Data Collection. This, with the intention to guarantee effective experiential learning. During Big Data Challenge, teachers and other mentoring figures actively engaged students in learning through group and project work. Finally, students conducted this learning activity in a period of about 1 month.

### The smart learning ecosystem

NSE was conceived and shaped accordingly with the third Master Plan (MOE, 2008) which aims to enrich and transform the learning environment to enable students to develop a critical digital expertise. NSE’s educational content was designed in such a way that learning activities were embedded in extra-curricular modules, minimizing interference with any scheduled school activities.

To do so, the major *government agency* (Fig. 1) involved during the implementation of the smart learning initiative was the Ministry of Education of Singapore who provided main linkages between NSE developers and educational institutes. In the same vein, the key *funding agency* was the National Research Foundation of Singapore, which is the authority that sets national directions for research and development by designing policies, plans, and strategies for research and innovation. In regard with *pedagogical institutes*, STEM Inc. helped delineating the learning agenda in form of Experiments. Partnerships with mentors from industry were also offered to schools, classes, and students with less experience in STEM subjects. The mentoring program helped bridging the gap between older and younger students.

The backbone of NSE’s smart learning ecosystem was built by r*esearchers and developers*. It was based on three ad hoc components: (i) SENSg, a wearable IoT device developed by Singapore University of Technology and Design (SUTD); (ii) An IoT cloud infrastructure (designed and operated by SUTD), and; (iii) ModStore, a web-based analytic tool for data analysis and visualization, implemented by the Singapore’s Institute of High Performance Computing (IHPC).

#### SENSg

**Fig. 3:**
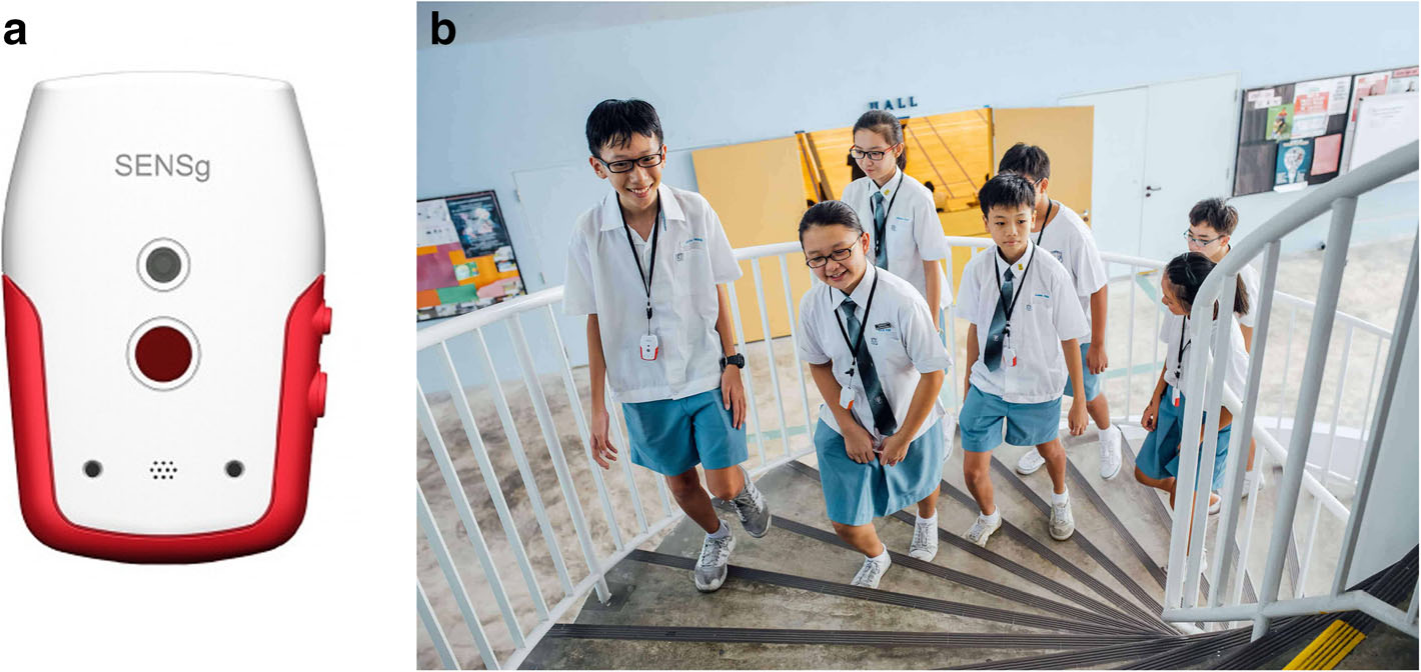
Sensor device and students during NSE

Its name stands for “Sense Singapore” and it can store multiple environmental, motion and location data at different sampling rates (Wilhelm et al., 2016). The Mode A (Mode B) of SENSg records raw data at rates of 1 reading every 13 s (5 readings every second). Using different sampling rates in delivering Data-driven Thinking in STEM education is important because higher sampling rates add computational and cognitive complexity (He, Ji, & Bobbie, 2017), thus, allowing elaborated designs of the learning environment. With a mass production of 50,000 SENSg devices, NSE simultaneously engaged a large number of schools, teachers and students. The top part of Table 1 reports the parameters and data recorded by SENSg (Fig. 3).

**Table 1:** List of sensors embedded in the SENSg device and other processed data

Sensor	Range	Accuracy	Units
*Raw data*			
Accelerometer	± 2 g ± 16 g	±(0.08–0.15)	g
Gyroscope	± 250 ± 2000	0.06	deg/sec
Magnetometer	± 4800	N/A	*μT*
Light Intensity	0.165 to 100 k	N/A	lux
Sound Pressure	30 to 130	SNR:63	dB
Relative Humidity	0 to 100	±3	%
Temperature	−10 to + 85	±0.3	° C
Pressure	300 to 1100	±0.12	hPa
IR Temperature	−40 to + 125	±3	° C
Button-press-event (happy moments)	–	–	Timestamp
*Processed data*			
Position	–	±100	m
Transportation mode	–	85	%
Number of steps	–	–	Integer
Transport/air conditioning CO_2_ emissions	–	–	Float
Access Point MAC addresses	–	–	–

#### IoT infrastructure

**Fig. 4:**
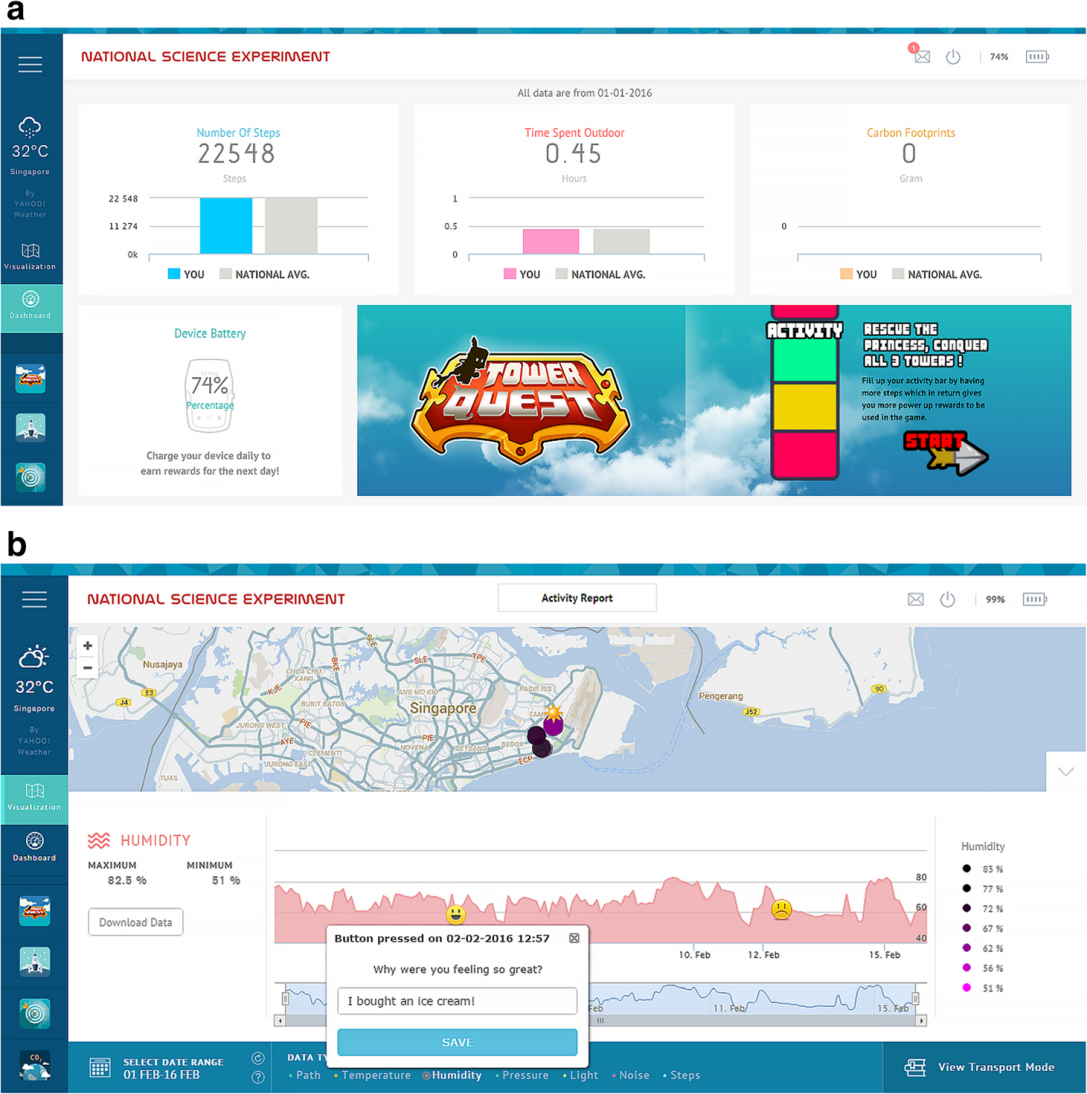
Dashboard and visualization page from the NSE web-app. **a** Dashboard of SENSg web-app displaying environmental and mobility data collected by the student. **b** Map with geo-located data points (top) and time series of a chosen parameter (bottom). Happy Moments are also shown with emojis characters, with the possibilities of adding comments to every single event (Benita et al., 2020)

After the data was collected, this was pushed and stored into NSE cloud servers. The infrastructure was designed to work at any time with all 50,000 SENSg devices active at once. Furthermore, the set up ensured out-of-school and off-line functionalities, e.g., students collecting data at any time in any place. We refer the interested reader to Wilhelm et al. (2016) for more details. After SENSg automatically pushed locally stored readings into main servers (once they went back to school), students had access to raw and processed data as shown in Table 1. Position refers to latitude and longitude geographic coordinates with the corresponding timestamp (developing spatial skills). Happy moments let students keep track of their moods (Benita, Bansal, & Tunçer, 2019). Transportation mode (Monnot et al., 2016; Monnot, Benita, & Piliouras, 2017; Wilhelm et al., 2017) distinguished between different means of transportation chosen by the student. The number of steps reported daily steps taken. CO_2_ emissions estimated daily emissions of carbon dioxide from transport and air conditioning usage (Happle, Wilhelm, Fonseca, & Schlueter, 2017). The above-mentioned processed data allowed students to be aware of energy saving and sustainable mobility. Additional elements of the IoT infrastructure were a website and a web-app (Fig. 4). The website showed guides, media and overall statistics while the web-app enabled interaction of students with SENSg (e.g., switching from Mode A to Mode B, or visualizing real-time readings). Additionally, by applying games as learning environments, the web-app was equipped with mini-games to foster the engagement of the youngest students.

#### Analytic platform: ModStore

**Fig. 5:**
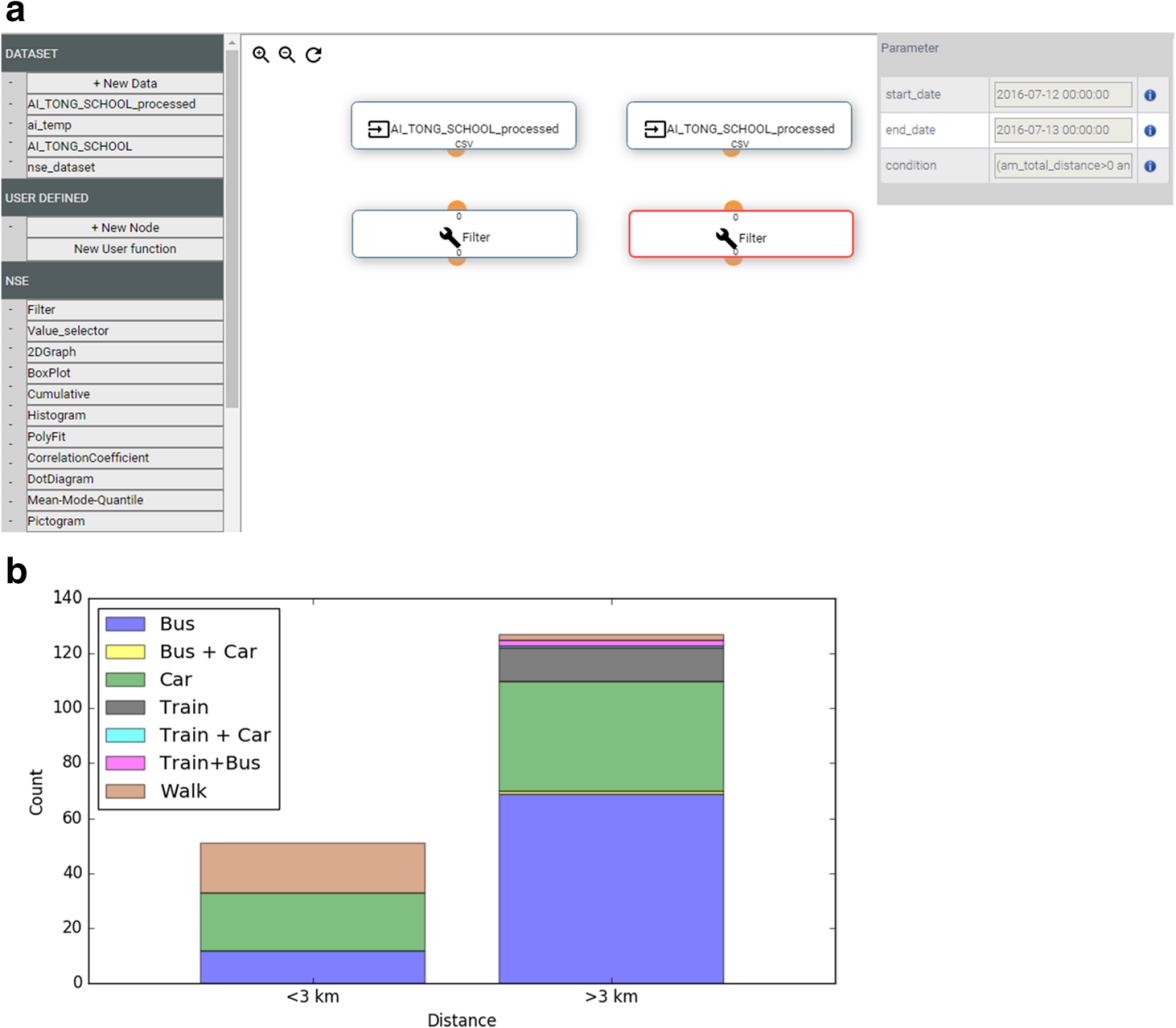
ModStore (Zhang et al., 2017). **a** Compositor to create workflows. **b** Most often used transport mode by distance traveled

It permitted students access and download their own data. It facilitated processing and data manipulation as it enabled students to perform analytical operations via simple algorithms and pseudo-code. The analytic platform was customized to follow relevant Ministry of Education math syllabus (Zhang et al., 2017). The engine is a browser-based software that allowed for the design of workflows (Fig. 5) in a drag-and-drop fashion (e.g., development of critical thinking, computational thinking and design thinking as detailed in Kitsantas and Dabbagh (2012), Wing (2006) or Grover and Pea (2013)).

## Results

Table 2 shows the “big” numbers of schools and students involved in the NSE smart learning initiative. The first NSE Experiment was launched in the last quarter of 2015 in the form of Data Collection 1. This stage was a major event for validating collaborations between stakeholders and functionality of the smart learning ecosystem when used by a large number of children and adolescent students. The engagement outputs of this stage were mainly measured by the total number of website visits and web-app users. Data Collection 2 was carried out during 2016 and promoted active learning by including the *happy button* which students were required to press whenever they felt happy.

**Table 2:** Participation of students during NSE

**Experiment**	**Schools**	**Students**	**Website visits**	**Web-app Users**
Data Collection 1	**129 Total**	**42,361 Total**	18,633	13,926
	67 (51.9%) Pri	23,691 (55.9%)		
	55 (42.7) Sec	16,993 (40.1%)		
	7 (5.4%) Pre-u	1,677 (4%)		
Data Collection 2	**93 Total**	**47,833 Total**	23,307	16,265
	41 (44%) Pri	13,364 (27.9%)		
	37 (39.8%) Sec	13,209 (27.6%)		
	15 (16.2%) Pre-u	21,260 (44.5%)		
			**Teams**	**Submitted Reports**
Big Data Challenge 1	**24 Total**	**235 Total**	58	44
	13 (52%) Sec	114 (48%)		
	12 (48%) Pre-u	121 (52%)		
Big Data Challenge 2	**45 Total**	**414 Total**	91	62
	34 (76%) Sec	280 (68%)		
	11 (24%) Pre-u	134 (32%)		

Primary school (Pri), Secondary school (Sec) and Pre-university (Pre-u)

Big Data Challenge 1 connected students with scientists from researcher and developer institutions to come up with innovative STEM applications by using the data collected during Data Collection 2. The connection between Data Collection periods and Big Data Challenges is that the former exposed students to get to track their carbon footprint, travel mobility patterns or amount of time they spend indoors/outdoors. Through Data Collection, students learned about IoT and Big Data while teachers were able to leverage the data to develop interesting physics lessons and teach concepts such as humidity, linear kinematics and pendulum motion through hypotheses testing and hands-on experiments.

The Big Data Challenges, gave students the freedom to create their own set of experiments, only constrained by the limitations of the SENSg device. Data Collections served as a step-stone to further exposing them to Data-driven Thinking through Big Data Challenges. In this stage teams of students (e.g., collaborative learning) were required to state a research question based on their own (schools’) data, perform analysis (using ModStore tool), develop and test hypotheses, draw meaningful insights, and to present their analyses in simple terms. Additionally, the instructional design of Big Data Challenge that included on-line tools ensured that participants who do not actively take part in the competition but stayed passive content consumers (so-called “lurkers”) could still benefit from participation (Ebner & Holzinger, 2005). In total, 58 teams from 24 schools participated in this challenge under two categories, which were Secondary schools and Pre-university, see Table 2. Among the addressed topics by the winners of this challenge in the Secondary schools’ category, we had: patterns of school commute, sleep and study; negative effects of transport and air-conditioning usage on carbon footprint; or the trade-off between schooling hours and sufficient duration of sleep. The topics explored by Pre-university students were more elaborated. For example, the importance of subjective well-being (i.e., happy moments) for mental and physical health; locations and attributes of most visited places; or the impact of traffic congestion on school starting times.

**Fig. 6:**
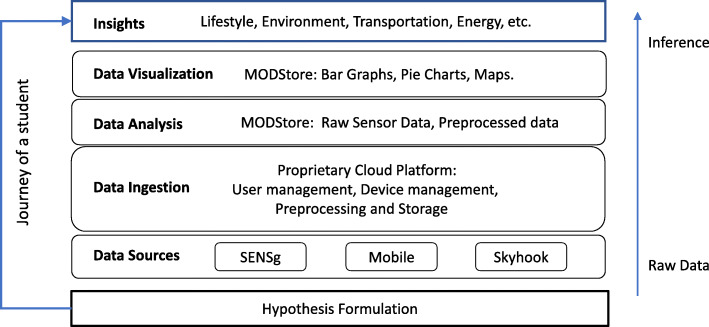
Representation of students’ performed activities during Big Data Challenge 2

The main difference between Big Data Challenge 1 and 2 is that in the latter, teams of students freely designed their own experiments (Fig. 6). Students were asked to think and formulate the hypothesis they wanted to test before moving to data collection through SENSg device or external datasets. Mentors from large companies such as IBM, Microsoft, Fujitsu, Delta Electronics, SAP, among others, were actively involved during the Big Data Challenge 2. Among the vast set of topics explored by students, winning teams investigated issues related to in- and out-of-school study patterns, CO_2_ emissions, preferences for physical activities, horizontal and vertical mobility, distribution of sleeping hours, comfort in the classrooms or noise propagation.

Final reports, column “Submitted Reports” in Table 2, were evaluated by experts during each Big Data Challenge, and competition-like setups of the Experiment were organized. The competition included prizes and awards to motivate students to actively participate and perform at their best. We refer the reader to the Appendix for details about differences in Data-driving Thinking gains derived from both Big Data Challenges.

## Discussion

### Concluding remarks

In this work, we have presented a general and scalable framework for designing, maintaining, and operating a smart learning ecosystem in STEM education. In doing so, all key stakeholders (educational institutions, pedagogical institutes, funding and government agencies, service providers, and researchers and developers) need to collaborate and concentrate efforts to ensure the success of the learning ecosystem. Moreover, our framework is characterized by Data-driven Thinking in the education process. To assure learning outcomes, elements of project-oriented problem-based learning, collaborative learning, experiential learning and gaming environments are adopted as core learning activities (Kolb, 2014; Morrison, Roth McDuffie, & French, 2015). Similarly, data plays a significant role in our learning framework and a plethora of (flexible) components are introduced, such as cloud computing, IoT devices or analytic platforms. We believe Data-driven Thinking will play a significant role in the future development of education systems (Coccoli, Guercio, Maresca, & Stanganelli, 2014; Grover & Pea, 2013; Ning & Hu, 2012; Tunçer, Benita, & Scandola, 2019), therefore, this paper contributes to the current understanding of the effective and efficient utilization of information technologies in the development of STEM education.

We have also shown through a case study how this smart learning ecosystem can be effective in practice. Our work describes the experience of Singapore’s National Science Experiment, the world’s largest Smart Education initiative where thousands of students and hundreds of teachers and staff got involved in an ecosystem that enabled Data-driven Thinking. Although the case study is based on Singapore, the proposed learning ecosystem and findings could have broad implications for other large cities with Smart Education initiatives worldwide. NSE is closely related to recent studies emerged from a variety of fields in STEM education. Using smartphones Cardone, Cirri, Corradi, and Foschini (2014) involved 300 students during 1 year in crowd sensing campaigns (ParticipAct) to incentive users to foster their participation in Smart Cities. In ParticipAct, students could voluntary decide to either accept or refuse to do requested activities, finding that only a minor number of students tried to provide fake data. Although the scope of the project was not directly based on educational outcomes, ParticipAct aligns with NSE in the aim to encourage residents to voluntary generate and provide data which can be of interest for public policymakers to optimize the available resources. In Hotaling (2009), the author carried out a three-years project (SENSE IT) with the goal of providing an infrastructure for teachers and students to design, implement and test student developed sensors. Implemented with 3000 high school and middle school students, SENSE IT challenged them to design, test, deploy and communicate with a set of (air temperature, conductivity, turbidity, and hydrostatic pressure) sensors. SENSE IT is probably the closest Smart Education initiative to NSE due to the aim of promoting STEM education in schools by offering an innovative learning experience through sensors. In the context of Smart Classroom, Gligorić, Uzelac, and Krco (2012) developed a real-time feedback on lecture quality tool to explore listener’s behavior in an intelligent environment. The use of IoT devices capturing video, sound, and infrared allowed the authors to improve classroom comfort levels. However, contrary to NSE, students were not actively involved during the experiment.

In nations lagging behind other countries in the fields of STEM, lessons learned from NSE, particularly the adoption of Data-driven Thinking, could provide a valuable knowledge base for the creation of (scalable) high-quality youth development programs. Children and adolescent students could have the opportunity to engage in scientific exploration and work together to build the next generation of scientists, engineers, and mathematicians. Methodologically speaking, our approach is opposite to traditional teaching model, which focuses on practice and remembering facts and procedures. On the other side of the spectrum, Data-driven Thinking encourages thinking and problem-solving as students can learn the importance of STEM subjects in everyday life, students’ interests, and concerns. On the basis of our findings, our recommendation for policy development is to focus on giving greater recognition to young students’ capabilities to engage with processes associated with the generation of ideas. Curriculum content should also emphasise the relevance of Project-oriented problem-based learning. Finally encouraging the generation, rather than the evaluation of ideas is way to foster STEM educational activities.

### Opportunities for STEM education in the face of COVID-19

The unprecedented times of COVID-19 have highlighted a new global need for remote learning in STEM areas where distance learning was not previously preferred. Educators have been forced to adapt course activities to accommodate online learning. The need of funding to acquire instructional materials, difficulties to (remotely) enforce assessment restrictions or limitations on the nature of the available e-learning tools (such as lifetime, functionality across different operating systems, efficiency, efficacy or satisfaction) are among the challenges faced by educational institutions and learners (Sintema, 2020; Van Nuland, Hall, & Langley, 2020). Our proposed smart learning framework may be helpful, if not essential, in creating additional remote course activities that ensure children and adolescents’ engagement. Moreover, our educational framework has been shown to ensure large-scale dissemination of Data-driven Thinking with tens of thousands of students. We have identified critical stakeholders together with their expected roles. Depending on the needs of the learners, educators and institutions, our ecosystem presents flexible learning opportunities and enables learners to learn synchronously (e.g., Data Collection) or asynchronously (e.g., Big Data Challenge) from a distance.

## Data Availability

Due to the nature of this research, participants of this study did not agree for their data to be shared publicly, so supporting data is not available.

## Appendix

### Competition

#### Big Data Challenge 1

Each team completing the Experiment had to prepare a final report and the submission was done via the EasyChair platform, a conference management system, to facilitate the evaluation procedures. Each report was evaluated by three experts from the operators of the NSE ecosystem (pedagogical institutes, service providers, researchers, and developers, see Fig. 1). The evaluation criteria included: (1) Innovation (novelty and/or originality); (2) Accuracy (error analysis); (3) Impact (findings and implications), and; (4) Presentation (quality of text and visualizations).

#### Big Data Challenge 2

Similar to Big Data Challenge 1, final reports were evaluated by three experts from the operators of the NSE ecosystem using the following criteria: (1) Research (problem identification, sources of information and problem analysis); (2) Solution (innovation, impact and technical accuracy); (3) Experiment (experimental plan, execution and error analysis), and; (4) Presentation (quality of text, quality of the visualizations and presentation effectiveness). Note that these judging criteria differs from the one used in Big Data Challenge 1 due to at this stage students were challenged to properly designed and conducted an experiment.

### Differences in Data-driving Thinking gains

A brief exploratory and inferential analysis of the student’s performance derived from their reports is presented in this section. The goal is to identify potential differences in learning outcomes during Big Data Challenge 1 and 2. The evaluation of Big Data Challenge 1 was carried out through a 100 points scale where each criterion (Innovation, Accuracy, Impact, and Presentation) was scored from 0 to 25. Report’s evaluation during the Big Data Challenge 2, in contrast, was done through a 5-point Likert scale (0–4), where each criterion (Research, Solution, Experiment, Presentation) was evaluated by 3 items described in the previous section. Although the scoring rubric was different in both years, it is possible to analyze differences on performance using non-parametric tests.

On the one hand, the Kruskal-Wallis post-hoc test for pairwise multiple comparisons allows us to identify factors that influence differences in scores. More precisely, we are interested in the test for each category (Secondary and Pre-university) H_0_(A): the evaluation criterion_*i*_ does not make a significant difference between the scores resulted from the reports. This is, the test allows us to explore if teams within the same category performed better/worse in a given criterion_*i*_. On the other hand, the Mann-Whitney U null hypothesis stipulates that two groups came from the same population. In other terms, we would like to test H_0_(B): the distribution of scores of criterion_*i*_ in Secondary school and Pre-university College categories are equal. The test helps us to understand if there is a differentiated effect in the learning process due to the student’s age.

Tables 3 and 4 summarize the findings, so that:

- Big Data Challenge 1.
  - H_0_(A): Applying the Kruskal-Wallis-post-hoc tests (after Nemenyi) shows there is no significant difference between the scores of Innovation, Accuracy, Impact and Presentation. This is true for both categories, Secondary school, and Pre-university.
  - H_0_(B): Applying the Mann-Mann-Whitney U test shows there is significant difference in scores of Innovation (*p*-value = 0.003) and Impact (*p*-value = 0.023) between Secondary school and Pre-university categories.
- Big Data Challenge 2.
  - H_0_(A): Applying the Kruskal-Wallis-post-hoc tests (after Nemenyi) shows there is significant difference in scores of the Solution criterion with respect to the rest of the criteria. This is true for both categories, Secondary school, and Pre-university.
  - H_0_(B): Applying the Mann-Mann-Whitney U test shows there is no significant difference in criteria scores between Secondary school and Pre-university.

**Table 3:** Differentiated effects of Data-driven Thinking, *p*-values. Big Data Challenge 1

H_0_(A): Difference between scores of various evaluation criteria									H_0_(B): Difference between scores
	Sec				Pre-u				
	Innovation	Accuracy	Impact	Presentation	Innovation	Accuracy	Impact	Presentation	Sec vs Pre-u
Innovation									**0.003**
Accuracy	0.33				0.96				0.12
Impact	0.99	0.51			0.73	0.41			**0.023**
Presentation	0.52	0.99	0.71		0.99	0.91	0.81		**0.02**

Secondary school (Sec) and Pre-university (Pre-u); *p*-value*<* 0.05 in bold font

**Table 4:** Differentiated effects of Data-driven Thinking, *p*-values. Big Data Challenge 2

H_0_(A): Difference between scores of various evaluation criteria									H_0_(B): Difference between scores
	Sec				Pre-u				
	Research	Solution	Experiment	Presentation	Research	Solution	Experiment	Presentation	Sec vs Pre-u
Research									0.45
Solution	**6.6E-06**				**0.04**				0.65
Experiment	0.99	**2.40E-05**			0.94	**0.008**			0.89
Presentation	0.89	**2.20E-04**	0.96		0.98	**0.18**	0.99		0.82

Secondary school (Sec) and Pre-university (Pre-u); *p*-value*<*0.05 in bold font

The exploratory analysis suggests that, during the Big Data Challenge 1, where students were limited to only perform analytics given fixed datasets and computational tools, teams of students within the same category (e.g., Secondary school or Pre-university) tended to achieve similar scores across all four criteria. However, teams of students from Secondary school category tended to perform lower in Innovation and Impact compared to Pre-university teams. The finding is expected, as Data-driven Thinking process was not yet met during the Big Data Challenge 1. Thus, more experienced teams of students tended to perform better.

Conversely, during Big Data Challenge 2 a differentiated performance on Solution criterion compared with Research, Experiment, and Presentation is found. In other words, both type of teams, Secondary school, and Pre-university, showed limitations in achieving promising insights derived from their experiments. This could be explained by the fact that Solutions criterion evaluates the last stage of the Data-driven Thinking, see Fig. 6, which may be the most difficult step to achieve. Moreover, most of the teams expressed a lack of time (about 3 weeks duration of Big Data Challenge 2) to obtain concluding findings. Some other teams reported issues during the data collection, affecting the quality of their final results whereas others informed that their dataset was too small to come up with concluding remarks. Interestingly, after delivering Data-driven Thinking experiences, there is no statistical evidence suggesting differences in the distribution of the criteria scores when comparing Secondary school vs Pre-university. In other words, both types of teams tended to perform equally well for any evaluated criteria. The finding is interesting as it shows that younger students tended to perform equally well as older students once the Data-driven Thinking framework was implemented.
